# Modification and Application Performance Study of Ultra-Fine Dry Powder Extinguishing Agent

**DOI:** 10.3390/molecules29163830

**Published:** 2024-08-12

**Authors:** Yurong Liu, Ruiyu Chen, Shuanglin Guo, Zhixuan Wang, Renming Pan

**Affiliations:** School of Safety Science and Engineering (School of Emergency Management), Nanjing University of Science and Technology, Nanjing 210094, China; 320103010109@njust.edu.cn (Y.L.); guo1755205739@163.com (S.G.); zx77.wang@foxmail.com (Z.W.)

**Keywords:** fluorocarbon structures, ultra-fine dry powder extinguishing agent, aviation firefighting, application performance

## Abstract

Ultra-fine dry powder extinguishing agent (UDPEA) is a promising alternative to Halon agents in aviation firefighting. The formulation of UDPEAs should balance environmental friendliness and practical engineering requirements, including high extinguishing efficiency, excellent flowability, and prolonged anti-reignition. This study investigates the effects of three modification methods (single perfluorooctyl triethoxysilane (FOTS), single N-(3-Triethoxysilylpropyl)perfluoro(2,5-dimethyl-3,6-dioxanonanoyl)amide (PFPE), and a combination of FOTS and PFPE at various mass ratios (2.0:0.4, 1.6:0.8, 1.2:1.2, 0.8:1.6, 0.4:2.0) (g)) on the performance of sodium bicarbonate-based UDPEA. The results indicate that using FOTS or PFPE alone improves the water and oil contact angles, but still fails to meet the required hydrophobicity and oleophobicity standards, and it also reduces the flowability and fire-extinguishing capability. A combination of FOTS and PFPE at the 1:2 ratio yields the best performance, with the water and oil contact angles of 145.169° and 143.542°, respectively, the lowest flowability index (0.224), minimal extinguishing concentration and time (14.183 g/m^3^ and 1.976 s, respectively), which is only 52.7% and 68.3% of those of the unmodified UDPEA’s (26.927 g/m^3^ and 2.893 s), and the longest anti-reignition time (68.5 s). In addition, the fire-extinguishing mechanisms (chemical inhibition and physical heat absorption) and anti-reignition mechanisms of the modified UDPEA (with the FOTS to PFPE ratio of 1:2) were revealed. This research aims to design an eco-friendly, high-performance UDPEA as an effective substitute for Halon extinguishing agents. These findings can provide valuable insights for evaluating and selecting aviation fire-extinguishing agents.

## 1. Introduction

Halon fire extinguishants have been widely used in aviation fire-suppression systems due to their high firefighting efficiency and cleanliness. However, because Halons contain chlorofluorocarbons, they contribute to ozone layer depletion and have a high greenhouse effect, raising concerns among environmental organizations and the international community. The Montreal Protocol of 1987 marked a significant milestone in global environmental protection by first proposing goals to phase out certain fluorinated hydrocarbon substances to protect the ozone layer [1]. Additionally, the 2016 “Kigali Amendment” demands the phased-out use of hydrofluorocarbons (HFCs) as fire-extinguishing agents, encompassing HFC-227ea, HFC-236fa, HFC-125, and so forth [2]. In pursuit of more environmentally friendly alternatives to Halon, scientists have shifted their focus to novel ultra-fine dry powder extinguishing agents (UDPEAs), known for their exceptional fire-suppression capabilities and full submergence characteristics [3].

However, UDPEAs still face several performance drawbacks in practical applications. Consequently, many researchers [4,5,6] have conducted studies to improve their performance. The following is a review of these deficiencies and the related research: Firstly, UDPEAs tend to agglomerate under high-temperature conditions, leading to decreased dispersion and flowability, which negatively affects extinguishing efficiency. To address this issue, Zhao et al. [7] designed a modified ammonium polyphosphate UDPEA with a C8 perfluorooctyl copolymer, which significantly enhanced its hydrophobic and oleophobic properties in water, diesel, aviation kerosene, and gasoline, thereby improving its high-temperature dispersion. Secondly, residual UDPEAs after firefighting may cause corrosion to metal components, such as those in aircraft, affecting the long-term service life of the equipment. Ning et al. [8] modified sodium bicarbonate UDPEA using silicone oil and a C6-fluorinated acrylic ester copolymer, resulting in a hydrophobic angle of 117° and an oleophobic angle of 120.5°. Building on Ning’s research, Liu et al. [9,10] further investigated the application performance (heat stability and corrosivity) of the modified UDPEA. Their results demonstrated that the modified UDPEA exhibited lower corrosiveness to aviation stainless steel alloy 0Cr18Ni9. Thirdly, some UDPEAs exhibit insufficient flowability during firefighting, failing to effectively cover the fire source, thus reducing firefighting efficiency. Research by Lu et al. [11] indicated that adding 6 wt.% hydrophobic fumed silica significantly improved the flowability and contact angle (126.48°) of the UDPEA. Additionally, adding 8% N-Hydroxysuccinimide to UDPEA resulted in minimal cohesive force, the highest flowability, and the lowest spray resistance, with a hydrophobic angle of 135.4° [12]. Finally, certain UDPEAs do not perform well in preventing reignition, failing to suppress fire over a long period, which poses safety risks. To enhance the anti-reignition performance of extinguishing agents, Zhang et al. [13] used perfluoropolyether methacrylate and N-(β-aminoethyl)-γ-aminopropyltrimethoxysilane to modify sodium bicarbonate UDPEA, significantly improving its anti-reignition time to 40.6 s for aviation kerosene.

It is worth noting that current research has predominantly focused on individual performance aspects of UDPEAs, with a notable lack of systematic optimization considering multiple performance criteria simultaneously. This narrow focus may result in improvements in one aspect while adversely affecting others. Future research should aim to comprehensively enhance various performance aspects—such as hydrophobicity, oleophobicity, flowability, and anti-reignition capability—while ensuring environmental friendliness to meet practical engineering application requirements. Previous improvements have partially addressed the performance deficiencies of UDPEAs in practical applications. However, the challenge remains to optimize multiple performance aspects concurrently. By rationally designing molecular structures, introducing appropriate modifiers, and systematically optimizing various performance criteria, it will be possible to develop more efficient and environmentally friendly UDPEAs.

In the modification research of UDPEAs, researchers [14,15] have designed low-surface-energy structures primarily using fluorocarbon chains with ≥8 carbon atoms. “C8”-type perfluorinated compounds (PFCs) are widely used due to their excellent hydrophobic and oleophobic properties and chemical stability. However, they are difficult to degrade and possess bioaccumulative and environmental pollution risks. In contrast, “C6”-type perfluorinated compounds, with their shorter molecular chains, exhibit better environmental friendliness. However, their weaker surface activity may lead to suboptimal modification effects, failing to form sufficiently stable and uniform coatings, which could impact the overall performance of the UDPEA. To address these issues, this study selected perfluoropolyether siloxane (PFPE) and 1H,1H,2H,2H-perfluorooctyltriethoxysilane (FOTS) for compounding. PFPE, unlike conventional “C9”-type perfluorinated compounds, is a polyether siloxane that includes multiple repeating perfluoroalkyl ether groups, giving it a more complex and flexible molecular structure. The polyether chains in PFPE provide molecular flexibility and some hydrophilicity, enhancing the dispersibility of the extinguishing agent in water and its spreading ability on various surfaces. Additionally, the -CF_3_ groups in PFPE significantly improve hydrophobic and oleophobic properties. Compared to other perfluorinated compounds, PFPE is more easily degraded in the natural environment, reducing its long-term presence and impact on the environment. The molecular structure of PFPE makes it difficult to accumulate in biological organisms, reducing potential toxicity and health risks associated with long-term exposure. FOTS, with its triethoxysilane groups, can form strong chemical bonds with substrate surfaces, increasing stability and durability, which helps the extinguishing agent maintain its performance in high-temperature fire environments. This combination aims to leverage the complementary advantages of PFPE and FOTS, addressing the deficiencies of “C6”- and “C8”-type compounds while ensuring enhanced environmental friendliness and performance of the modified UDPEA.

Therefore, this study chose to modify sodium bicarbonate UDPEAs using three different approaches: single PFPE, single FOTS, and a combination of PFPE and FOTS. The aim is to investigate the application performance of the modified UDPEAs, including their microscopic morphology, chemical structure, fluidity, hydrophobicity and oleophobicity, extinguishing concentration and time, and anti-reignition time, under different formulations. By understanding the relationship between formulation and performance, the goal is to harness the multiple advantages of their molecular structures and develop an ideal material that achieves the optimal balance between environmental friendliness and performance. This research aims to design an eco-friendly, high-performance extinguishing agent and provide a design method for an eco-friendly, high-performance UDPEA, thereby making a positive contribution to the replacement of Halon extinguishing agents.

## 2. Materials and Methods

### 2.1. Sample Preparation

**Figure 1:**
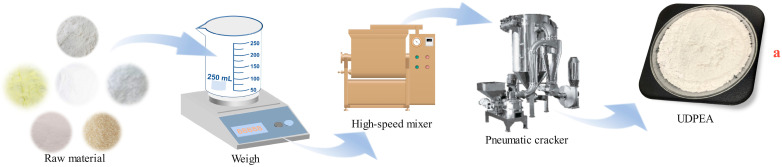
The preparation process of UDPEA.

The dry powder extinguishing agent (DPEA) was prepared with sodium bicarbonate, mica powder, talcum powder, activated clay, zeolite, and nano-calcium carbonate (Nanjing Chemical Reagent Co., Ltd., Nanjing, China) in a ratio of 90:2.7:2.7:1.35:1.9:1.35. After thorough mixing with a high-speed mixer, the DPEA was further micronized by an air jet mill to achieve a D90 (the particle size at which 90% of the particles are smaller) of less than 10 μm, obtaining sample “a”. The preparation process is illustrated in Figure 1.

**Figure 2:**
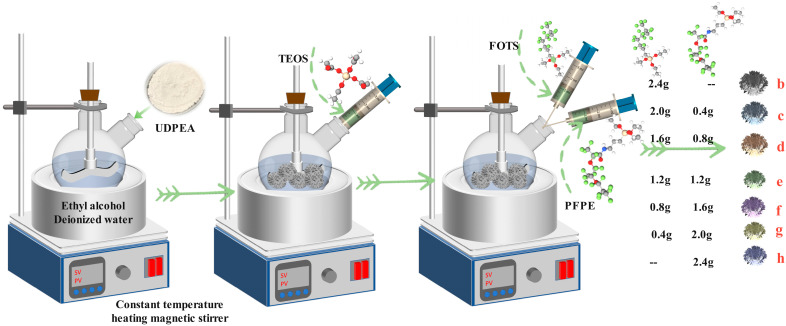
The preparation process of MUDPEAs.

Next, 140 mL ethanol and 10 mL deionized water were added to a three-neck flask. The mixture was stirred at 300 r/min for 5 min using a mechanical stirrer. Subsequently, 36.0 g of sample “a” was added to the three-neck flask and stirred at room temperature for 2 h. Then, 2.4 g tetraethyl orthosilicate (TEOS) was added to the three-neck flask and stirred at room temperature for 4 h. Subsequently, the following were added separately to the flask: 2.4 g FOTS; 2.0 g FOTS and 0.4 g PFPE; 1.6 g FOTS and 0.8 g PFPE; 1.2 g FOTS and 1.2 g PFPE; 0.8 g FOTS and 1.6 g PFPE; 0.4 g FOTS and 2.0 g PFPE; and 2.4 g PFPE. The mixture was stirred at room temperature for 12 h in the three-neck flask. The resulting solid-liquid mixture was filtered, and the powder was dried at 60 °C for 10 h and then ground to obtain modified UDPEAs samples “b”, “c”, “d”, “e”, “f”, “g”, and “h”. The preparation process is illustrated in Figure 2. Detailed functions of each auxiliary additive are shown in Appendix A.

### 2.2. Characterization

The microstructure of the samples was examined using the JSM-IT500HR scanning electron microscope from JEOL from Tokyo, Japan. The electron microscope was operated at a working voltage of 15 kV, with samples coated with a thin layer of gold to enhance conductivity. Particle size distribution was measured using the Mastersizer 3000 laser particle size analyzer from Malvern Instruments Ltd from Darwin, England. The dispersant used was pure water, and samples were sonicated for 3 min to ensure uniform dispersion. The measurement range was from 0.01 to 3500 μm. The chemical structure of the powder was analyzed using the NICOLET iS10 Fourier transform infrared spectrometer from Waltham, MA, USA, with a scan range of 4000–400 cm^−1^, a resolution of 4 cm^−1^, and each sample scanned 32 times. X-ray diffraction analysis was performed using the Rigaku D/max-2500pc X-ray diffractometer from Rigaku, Japan, with Cu Kα radiation (λ = 1.5406 Å). The scan range was from 5° to 90°, with a step size of 0.02° and a scanning speed of 1°/min.

In order to evaluate the flowability of the powder, the tapped density of the powder was measured using the HY-100 A powder vibration densitometer from DanDong, China. The *N*/*C* ratio (number of vibrations divided by the number of compressions) is a linear function of the vibration number (*N*), as shown in Equation (1). The reciprocal of the slope (*l_p_*) represents the flowability index of the tested powder, while *f_a_* denotes the adhesion force of the measured powder particles. The smaller *l_p_* and *f_a_* value indicate better flowability.

(1) $$N/C=\frac{1}{l_{p}}\cdot N+\frac{1}{l_{p}}\cdot f_{a}$$

**Figure 3:**
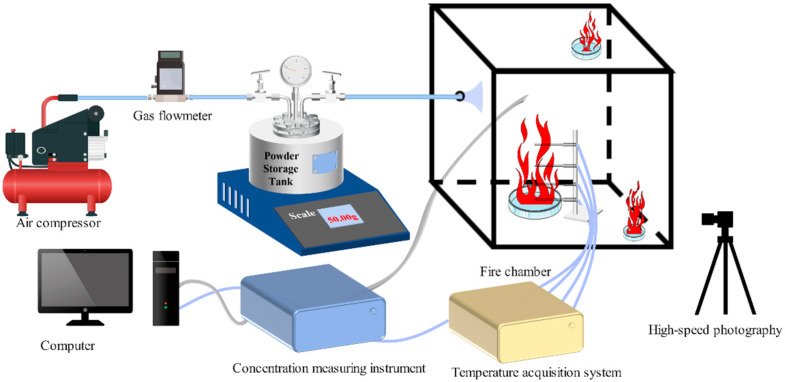
Fire-extinguishing experiment in a 1 m^3^ cubic chamber.

The fire-extinguishing efficiency of the powder was evaluated using a 1 m^3^ cubic fire-extinguishing chamber, as shown in Figure 3. In the central area, a circular steel oil pan with the diameter of 0.15 m was placed. Four thermocouples were positioned along the vertical axis above the oil pan at 0.08 m intervals. The autonomously developed automatic ignition procedure was employed: Upon activation via a mouse click, temperature collection began immediately, followed by automatic ignition after a 1-s delay. A pre-burn period of 119 s was observed before initiating concentration data collection. After a subsequent 0.5-s delay, the high-speed cameras were activated, followed by another 0.5-s delay before the electromagnetic valve was triggered to release the pre-loaded powder from the storage tank. Each sample underwent three separate fire-extinguishing trials; failure to extinguish the fire in any of these trials was deemed an overall failure for the sample. To mitigate incidental errors, the highest extinguishing concentration value among the three trials was selected as the sample’s extinguishing concentration.

In order to evaluate the hydrophobicity, oleophobicity, and anti-reignition performance of the powder, the contact angles [16,17] between the sample and water and oil were measured using an SDC-100 contact angle measuring instrument from Dongguan, China. After spreading the fire-extinguishing agent on the surface of the oil, the settling of the fire-extinguishing agent above the oil surface was observed. The oil was continuously ignited until re-ignition occurred for the second time, and the anti-reignition time was recorded.

## 3. Results and Discussion

### 3.1. Microscopic Morphology Analysis

**Figure 4:**
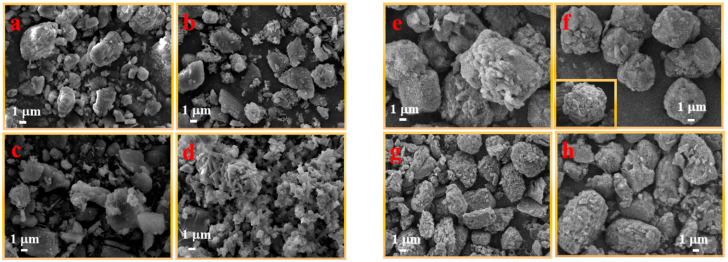
The microscopic morphology of samples: (**a**) UDPEA, (**b**) UDPEA modified by FOTS, (**c**) UDPEA modified with FOTS and PFPE at a mass ratio of 5:1, (**d**) UDPEA modified with FOTS and PFPE at a mass ratio of 2:1, (**e**) UDPEA modified with FOTS and PFPE at a mass ratio of 1:1, (**f**) UDPEA modified with FOTS and PFPE at a mass ratio of 1:2, (**g**) UDPEA modified with FOTS and PFPE at a mass ratio of 1:5, (**h**) UDPEA modified by PFPE.

Figure 4 shows the micro-surface morphology of UDPEA (a) and modified UDPEA (b–h). The average diameter of UDPEA particles is about 6 μm, as shown in Figure 4a, with a scattering of more auxiliary small particles around the sodium bicarbonate. After modification using FOTS and TEOS, as shown in Figure 4b, a fluffy layer adhered to the surface of the sodium bicarbonate, with fewer scattered auxiliaries, and the particles exhibited irregular shapes. Figure 4h depicts the micro-surface morphology of UDPEA after modification with PFPE and TEOS. Compared with that shown in Figure 4b, its micro-surface morphology is more elliptical. This may be due to the stronger hydrophilicity and the larger intermolecular forces of the FOTS molecular groups, leading to the easier formation of spherical structures during the crystallization or aggregation process. The molecular structure of PFPE may tend to exhibit sharp or irregular forms in the formation of particles. Figure 4c–g shows the micro-surface morphology of modified UDPEAs with different ratios of FOTS and PFPE. As the proportion of PFPE to FOTS increases, the modified UDPEA particle gradually transforms from irregular polyhedral shapes to spherical shapes. When the ratio of PFPE to FOTS reaches 2:1, as shown in Figure 4f, the micro-surface morphology exhibits regular spherical particles, and the powder surface is covered with a uniform fluffy hydrophobic and oleophobic layer, with the particle size and shape becoming basically consistent. As the ratio of PFPE to FOTS further increases, the powder gradually transforms into ellipsoids. This indicates that the interaction between FOTS and PFPE may lead to changes in crystal morphology, ultimately resulting in adjustments to the powder morphology. The molecular structure of PFPE may make it easier to form ellipsoidal particles during crystal growth. When mixed with FOTS, the spherical morphology of PFPE gradually emerges, causing the overall powder morphology to tend toward spherical shapes.

### 3.2. Chemical Structure Analysis

**Figure 5:**
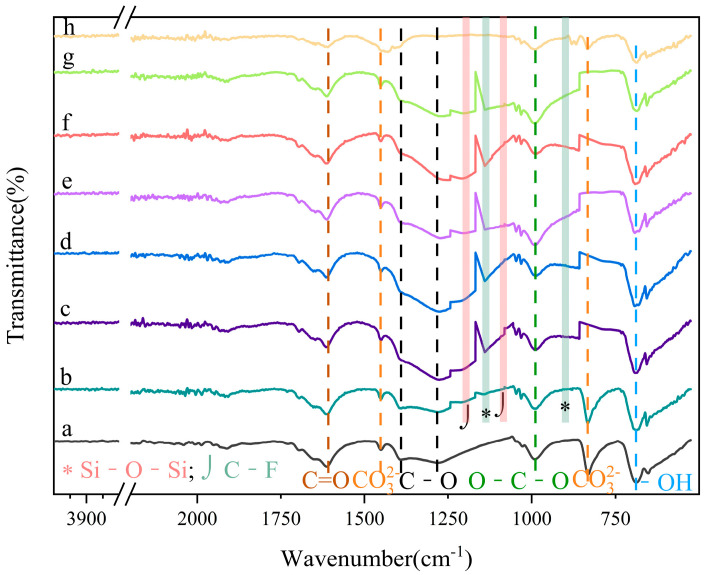
The infrared spectra of samples: (**a**) UDPEA, (**b**) UDPEA modified by FOTS, (**c**) UDPEA modified with FOTS and PFPE at a mass ratio of 5:1, (**d**) UDPEA modified with FOTS and PFPE at a mass ratio of 2:1, (**e**) UDPEA modified with FOTS and PFPE at a mass ratio of 1:1, (**f**) UDPEA modified with FOTS and PFPE at a mass ratio of 1:2, (**g**) UDPEA modified with FOTS and PFPE at a mass ratio of 1:5, (**h**) UDPEA modified by PFPE.

Figure 5a shows the infrared spectrum of UDPEA, with characteristic absorption peaks at 1609 cm^−1^, 1449 cm^−1^, 1394 cm^−1^, 1274 cm^−1^, 988 cm^−1^, 687 cm^−1^, and 652 cm^−1^ representing the sodium bicarbonate [10]. The features at 1609 cm^−1^ and 1274 cm^−1^ are attributed to the stretching vibrations of the C=O and C-O functional groups in NaHCO_3_ [18]. The infrared absorption peaks at 988 cm^−1^ and 652 cm^−1^ are attributed to the deformation vibration absorption of the O-C-O group and the bending vibration absorption of the O-H bond in the bicarbonate ion, respectively [19]. The peaks at 1449 cm^−1^, 1394 cm^−1^, and 829 cm^−1^ correspond to the asymmetric stretching, symmetric stretching vibration, and bending vibrations of the CO_3_^2−^ group [20], indicating that the basic chemical structure of the main component—namely, sodium bicarbonate—in UDPEA has not changed. In the FT-IR spectra of the modified UDPEAs, the peaks at 1203 cm^−1^ and 1151 cm^−1^ are caused by the stretching vibrations of C-F in the -CF_2_ and -CF_3_ groups [21,22]. This indicates that PFPE hydrolyzes and coats the surface of the powder, exposing the groups containing -CF_2_ and -CF_3_. Additionally, the peaks at 1074 cm^−1^ and 878 cm^−1^ are attributed to the asymmetric stretching and bending vibrations of the Si-O-Si bond in the silane coupling agent TEOS [23]. Therefore, the typical infrared absorption peaks of the C-F and Si-O bonds in the seven types of modified UDPEAs (b–h) indicate the successful sol-gel hydrolysis reaction of fluorosilane and TEOS on the surface of NaHCO_3_.

**Figure 6:**
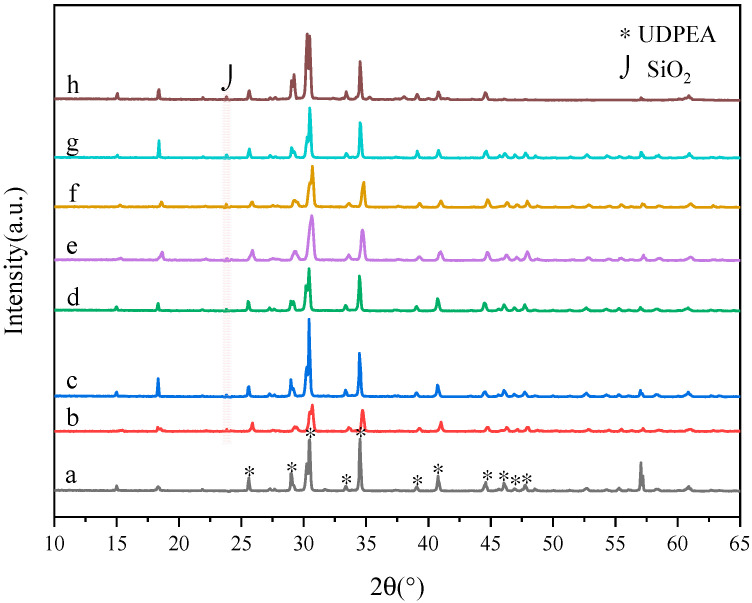
XRD diffraction spectra of samples: (**a**) UDPEA, (**b**) UDPEA modified by FOTS, (**c**) UDPEA modified with FOTS and PFPE at a mass ratio of 5:1, (**d**) UDPEA modified with FOTS and PFPE at a mass ratio of 2:1, (**e**) UDPEA modified with FOTS and PFPE at a mass ratio of 1:1, (**f**) UDPEA modified with FOTS and PFPE at a mass ratio of 1:2, (**g**) UDPEA modified with FOTS and PFPE at a mass ratio of 1:5, (**h**) UDPEA modified by PFPE.

Figure 6 presents the X-ray diffraction (XRD) spectra of eight samples. In Figure 6a, the diffraction peaks marked with an asterisk (*) are the characteristic peaks of UDPEA. Figure 6b–h demonstrates that modifying the surface of sodium bicarbonate with fluorosilane does not alter the crystalline form of NaHCO_3_ [24]. The characteristic diffraction peaks of SiO₂ are not prominent in the spectra of the modified UDPEAs. However, the broad diffraction peak around 2θ = 23° corresponds to the amorphous SiO_2_ diffraction peak. This indicates that the SiO₂ generated by the hydrolysis of TEOS and fluorosilane is in a typical disordered amorphous state [25]. The presence of SiO₂ diffraction peaks confirms the successful hydrolysis of TEOS and the coating of sodium bicarbonate with the modification agent (PFPE and FOTS).

### 3.3. Flowability Analysis

**Figure 7:**
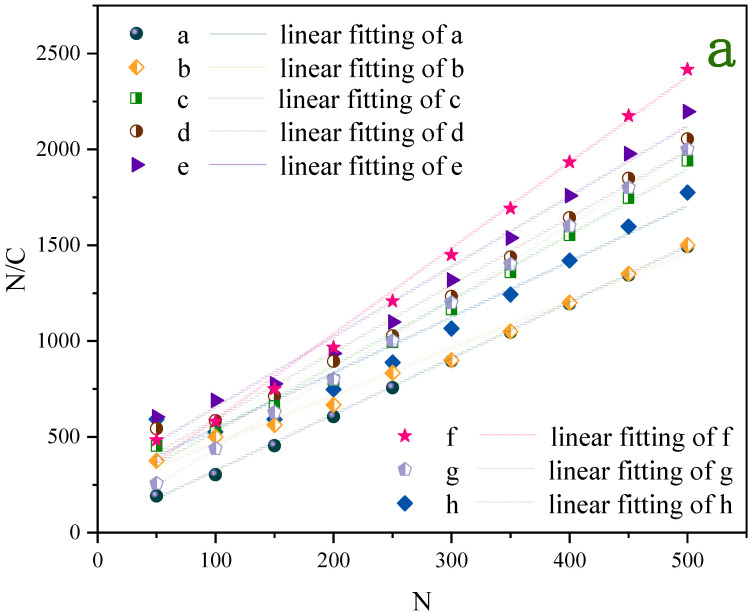

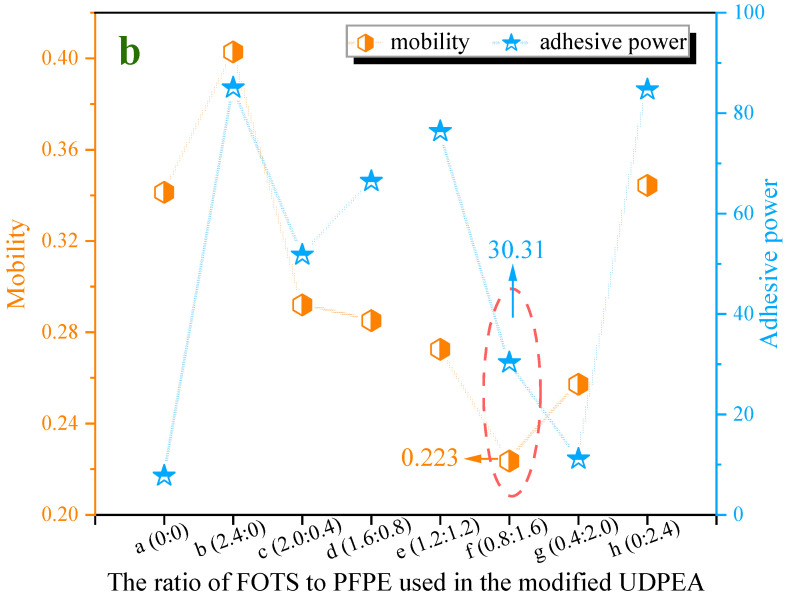
(**a**) The relationship between compressibility and vibration frequency for samples a–h, and (**b**) the flowability parameters.

High flowability allows the UDPEA to spread more evenly over the fire source. This uniform coverage improves the effectiveness of the extinguishing agent by ensuring that all areas of the fire are addressed, thus enhancing extinguishing efficiency. To investigate the changes in flowability performance of UDPEA and UDPEA modified with single FOTS, single PFPE, and FOTS and PFPE at mass ratios of 5:1, 2:1, 1:1, 1:2, and 1:5, respectively, we measured the compressibility (*N*/*C*) data of samples a–h under different vibration frequencies (*N*). Detailed data can be found in Table S1 in the Supplementary Material. The relationship between compressibility and vibration frequency for samples a–h is shown in Figure 7a. The compressibility versus the number of vibrations for samples “a”, “b”, “c”, “d”, “e”, “f”, “g”, and “h” conform to the linear equations N/C=2.93N+22.69, N/C=2.90N+245.89, N/C=3.67N+280.21, N/C=3.51N+233.09, N/C=3.42N+177.32, N/C=4.47N+135.56, N/C=2.48N+211.11, N/C=3.89N+43.31, respectively, with R² values all greater than 0.990.

As observed in Figure 7b, the flowability index (*l_p_*) and adhesion index (*f_a_*) of UDPEA are 0.341 and 7.74, respectively. When sole FOTS or PFPE are used to modify the UDPEA, represented by samples “b” and “h”, their flowability indices are 0.403 and 0.344, increasing by 18.2% and 0.9% compared to that of sample “a”, respectively. The corresponding adhesion indices are 80.05 and 84.68, which are 9.3 and 9.9 times higher than that of sample “a”, respectively. This indicates that the introduction of either FOTS or PFPE alone can form a critical stable structure in the microstructure of the UDPEA. This structure has strong cohesion, enhancing the interactions between particles and thus increasing adhesion. The enhanced particle interactions hinder the free movement of particles, leading to decreased flowability. In addition, PFPE may form a relatively complex film on the particle surface, making it smoother and having a negligible impact on flowability, as shown in Figure 4b,h.

Specifically, as the ratio of FOTS to PFPE decreases, the flowability index of the modified UDPEAs (samples “c”, “d”, “e”, “f”, “g”) first decreases and then increases. This indicates that the flowability of the modified UDPEAs initially improves and then deteriorates with the decreasing ratio. Simultaneously, the adhesion index shows an initial increase followed by a decrease. When the ratio of FOTS to PFPE is 1:2, the flowability index of sample “f” reaches its lowest value (0.224). This might be because when the ratio of FOTS to PFPE is 1:2, the relatively higher PFPE content ensures that more hydrophobic and oleophobic groups (-CF_3_ and -CF_2_) are exposed on the modified UDPEA surface. The -CF_3_ and -CF_2_ groups in PFPE not only exhibit strong hydrophobic properties but can also interact with the FOTS-modified surface through van der Waals forces, forming stable Si-O-Si bonds. This bonding not only ensures the stability of the modification layer but also provides a synergistic effect with the PFPE molecular chains, making it easier to uniformly coat the powder surface. Under this ratio, the synergistic effect of FOTS and PFPE is maximized, ensuring the chemical stability of the modification layer while maximizing hydrophobicity, oleophobicity, and fluidity. Modified UDPEA likely forms a more uniform and ideal wetting layer on the surface, enhancing particle interactions and thus improving both flowability and adhesion. This optimization is significant for the effective dispersion of the fire-extinguishing agent.

### 3.4. Analysis of Hydrophobic and Oleophobic Performance

**Figure 8:**
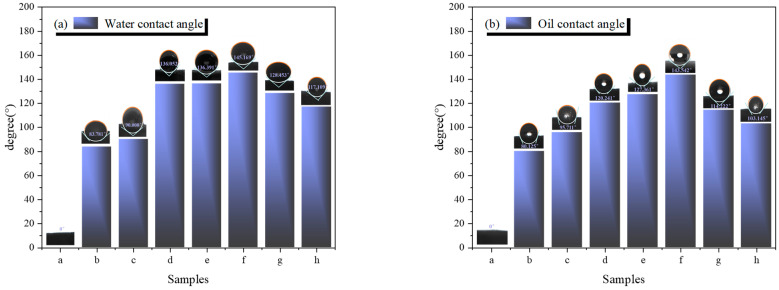
(**a**) Water contact angle and (**b**) Oil contact angle of samples a–h.

Figure 8 shows the hydrophobic and oleophobic angles of samples a–h. When deionized water or aviation kerosene is dropped onto the surface of UDPEA, it quickly disappears, indicating that UDPEA lacks hydrophobic and oleophobic properties. After modifying UDPEA with FOTS and TEOS, the water and oil contact angles of sample b are 83.781° and 80.125°, respectively, both less than 90° (A 90° angle is often used as a reference point to assess whether a material exhibits neutral wettability), showing that sample “a” is hydrophilic and oleophilic.

When FOTS and PFPE are combined at the ratio of 5:1 and used to modify UDPEA, the water and oil contact angles of sample “c” are 90.000° and 95.711°, respectively. Compared to sample “b”, which is modified solely with FOTS, the fire-extinguishing agent changes from hydrophilic and oleophilic to hydrophobic and oleophobic. This transformation is likely due to the hydrophobic nature of FOTS, which is attributed to the presence of fluorine atoms, resulting in the hydrophobicity of the modified sodium bicarbonate. Furthermore, PFPE, containing long perfluoroalkyl chains, is a fluorinated polymer with excellent oleophobic properties. In sum, by copolymerizing FOTS and PFPE at specific ratios, the resulting composite material combines the hydrophobicity of FOTS with the oleophobicity of PFPE, making the modified sodium bicarbonate both hydrophobic and oleophobic. The combined effect of hydrophobic FOTS, oleophobic PFPE, and the silica coating formed by TEOS alters the surface properties of sodium bicarbonate, imparting both hydrophobic and oleophobic characteristics.

As the ratio of FOTS to PFPE decreases, both hydrophobicity and oleophobicity first increase and then decrease. For sample “f”, as shown in Figure 8, when FOTS and PFPE are combined at the ratio of 1:2, the hydrophobic and oleophobic angles reach their maximum values of 145.169° and 143.542°, respectively. This indicates that at this ratio, there might be a synergistic effect between FOTS and PFPE, enhancing the material’s hydrophobicity and oleophobicity more than in other cases.

In addition, the excellent hydrophobic and oleophobic properties can reduce the surface energy of the powder. As shown in Figure 4f, the fire-extinguishing agent particles are spherical, and a uniform velvet-like layer can be observed on the surface, effectively preventing the infiltration of water and oil, thereby forming the observed high contact angle. Hydrophobic and oleophobic groups such as -CF_3_ and -CF_2_ can form a lubricating layer on the particle surface, reducing the friction coefficient between the powder particles [26,27], which also explains the significantly improved flowability of sample “f”, as verified in Section 3.3.

### 3.5. Fire-Extinguishing Performance Analysis

**Figure 9:**
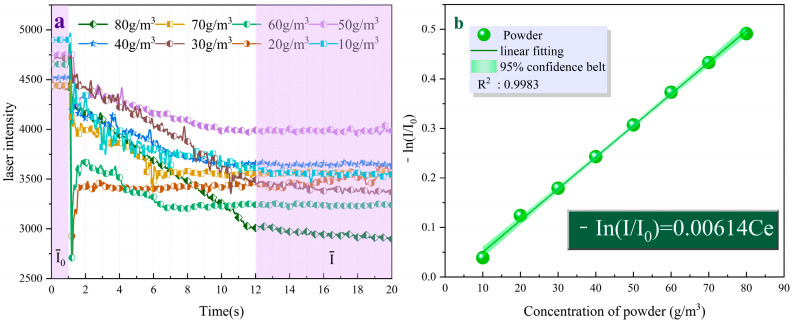
(**a**) Variations of light intensity over time for UDPEA at different concentrations and (**b**) Negative logarithm of laser absorbance of the UDPEA as a function of concentration.

**Figure 10:**
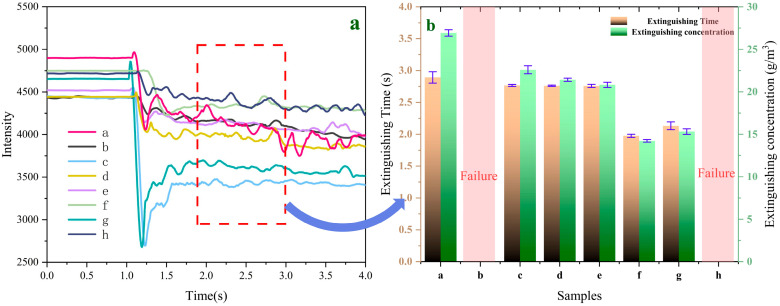
(**a**) The time-varying curves of laser intensity during the fire-extinguishing process for samples a–h and (**b**) Extinguishing time and extinguishing concentration of samples a–h for aviation kerosene fires.

Figure 9a shows the variation of light intensity over time for the UDPEA at different concentrations. Upon fitting, the negative logarithm of the ratio of laser intensity after the uniform distribution of the UDPEA in space to the initial light intensity is proportional to its concentration, as described by Equation (2). The correlation coefficient value R² is greater than 0.998, indicating a high linear correlation:

(2) $$-\ln(I/I_{0})=0.00614C_{e}$$

where, *I*_0_ and *I* denote the initial light intensity and the light intensity after the fire-extinguishing agent concentration reaches a constant value, and *C_e_* is the concentration of the fire-extinguishing agent. Since fire-extinguishing performance is a crucial indicator for evaluating fire-extinguishing agents, samples a–h were used in fire-extinguishing tests in a 1 m^3^ fire testing chamber. Figure 10a,b shows the variation curves of laser intensity over time during the fire-extinguishing process and the time and concentration required to extinguish aviation kerosene fires for samples a–h, respectively. According to Figure 10b, the extinguishing time and concentration of sample “a” are 2.893 s and 26.927 g/m^3^, respectively.

Using UDPEA’s extinguishing time and concentration as benchmarks, experiments show that samples “b” and “h”, loaded with the same mass (30.0 g) as UDPEA, failed to successfully extinguish aviation kerosene fires. Weighing the powder storage tanks revealed no residual UDPEA, whereas 12.3 g and 10.8 g of samples “b” and “h”, respectively, adhered to the walls and bottom of the tanks. This indicates that under the same driving pressure, the amount of sample “b” and “h” carried by the gas was less than that of UDPEA. This is because when only PFPE or FOTS is used to modify UDPEA, the flowability index (l_p_) and adhesion (*f_a_*) of the samples “b” and “h” significantly increase, reducing its flowability and enhancing its adhesion, as shown in Figure 7b. Therefore, using samples “b” or “h” would require additional mass, which does not meet the aircraft weight-reduction requirements. However, when FOTS and PFPE are combined to modify UDPEA, the extinguishing time and concentration first decrease and then increase with the decreasing ratio of FOTS to PFPE. Especially, when the ratio is 1:2, sample “f” achieves the lowest extinguishing concentration of 14.183 g/m^3^, which is only 52.7% of that of UDPEA. This means that under the same conditions, the amount of sample “f” required to extinguish an aviation kerosene fire is only about half of that needed for UDPEA, which is crucial for weight reduction in aircrafts. Moreover, the extinguishing time for sample “f” is 1.976 s, which is 31.7% less than that of UDPEA, significantly improving the speed of fire control.

### 3.6. Anti-Reignition Analysis

**Figure 11:**
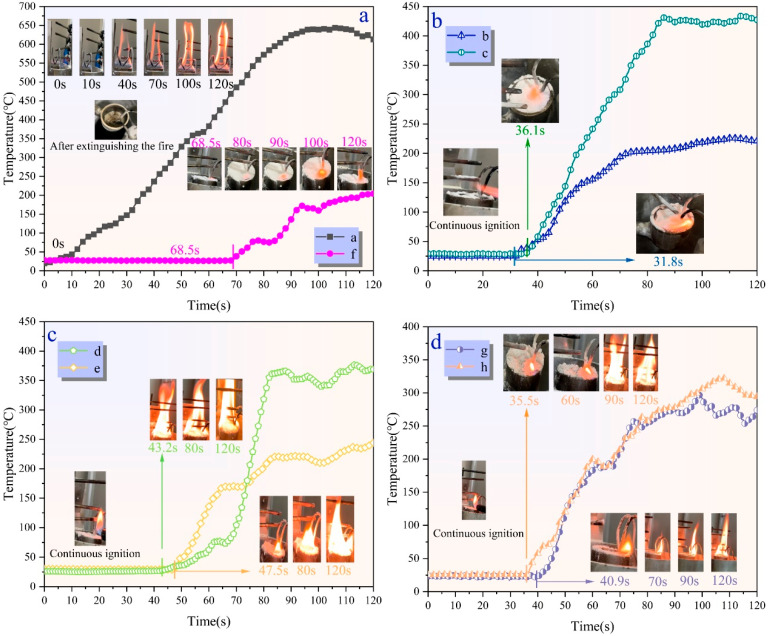
Anti-reignition temperature variation over time: (**a**) samples “a” and “f”, (**b**) samples “b” and “c”, (**c**) samples “d” and “e”, (**d**) samples “g” and “h”.

Figure 11 shows the anti-reignition temperature variation over time for samples a–h. As shown in Figure 9a, sample “a” could not float on the surface of aviation kerosene. After extinguishing the fire, when the oil surface was reignited, it immediately re-ignited, and after burning for 120 s, the oil surface temperature exceeded 650 °C, with intense combustion observed. At the end of the experiment, significant deposition of UDPEA powder was observed at the bottom of the burning cup, indicating that conventional UDPEA lacks anti-reignition properties. In real fire scenarios, if extinguished oil fires encounter another ignition source, the previously sprayed UDPEA cannot provide help for anti-reignition.

Based on the microstructure analysis, flowability analysis, and hydrophobicity and oleophobicity properties analysis in Section 3.1, Section 3.3 and Section 3.4, sample “f” probably has the best anti-reignition properties. As observed in Figure 11a, aviation kerosene covered by sample “f” showed weak flames on the powder surface after continuous ignition for 68.5 s, with the temperature rising slowly. At 120 s, only a tiny part of the oil surface was ignited, with no significant fire spread, and the temperature remained below 200 °C.

Figure 11b,c shows that the anti-reignition time for samples “b”, “c”, “d”, and “e” is 31.8 s, 36.1 s, 43.2 s, and 47.5 s, respectively. At 120 s, the flame temperatures ranged from 220 °C to 450 °C, which was significantly lower than the flame temperature of sample “a”. However, the anti-reignition times are only 46.4% to 69.3% of that of sample “f”. As seen in Figure 11d, the anti-reignition time for samples “g” and “h” is 40.9 s and 35.5 s, respectively, with temperature around 300 °C at 120 s.

Experimental results indicate that the modification of UDPEA with either FOTS or PFPE alone can impart some anti-reignition to UDPEA, but the anti-reignition time remains relatively short. When FOTS and PFPE are combined at specific ratios, the anti-reignition is improved significantly. As the ratio of FOTS to PFPE decreases, the anti-reignition time first increases and then decreases. Specifically, when the ratio is 1:2, the anti-reignition time reaches its maximum value (68.5 s), and the oil surface temperature at 120 s is the lowest. This suggests that in the actual oil fires, spraying sample “f” can effectively suppress or delay anti-reignition, which is crucial for secondary rescue operations.

### 3.7. Fire-Extinguishing and Anti-Reignition Mechanism

**Figure 12:**
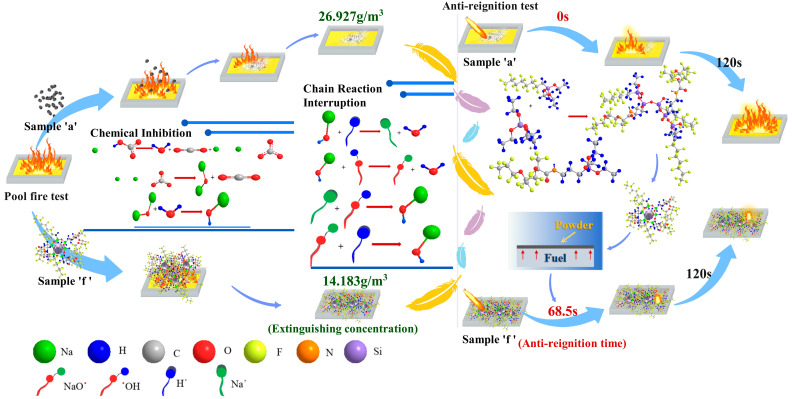
Fire-Extinguishing and Re-ignition Resistance Mechanisms of Samples “a” and “f”.

Based on the analysis in Section 3.1, Section 3.2, Section 3.3, Section 3.4 and Section 3.5, sample “f” exhibits the best comprehensive performance. Figure 12 illustrates the fire-suppression and anti-reignition mechanisms of sample “f” compared to the conventional UDPEA (sample “a”). The active component of both UDPEA and modified UDPEA is sodium bicarbonate, which rapidly decomposes upon contact with flames to produce sodium carbonate, water, and inert gas carbon dioxide. Sodium carbonate further decomposes at high temperatures into sodium oxide and carbon dioxide, with sodium oxide reacting with water to form sodium hydroxide. During this process, UDPEA and modified UDPEA extinguish fires through heat absorption and oxygen isolation.

Studies have shown that in actual fire scenes, heat is often released in the form of electromagnetic waves to promote fuel evaporation [28]. Notably, when modified UDPEA enters the fire scene, the powder disperses around the flames in an atomized form, creating a mist cloud. Its surface is covered with a fluffy hydrophobic and oleophobic layer, which increases the specific surface area of the particles and enhances heat absorption. Simultaneously, the superior flowability of modified UDPEA (l_p_ = 0.224) allows it to flow smoothly during spraying and make full contact with the flames quickly. This effective absorption of radiant heat is crucial for fire suppression as it blocks heat transfer between the fire source and combustible materials.

Previous studies have found that the thermal decomposition temperature of modified UDPEA is delayed [10], and its thermal stability is significantly improved compared to UDPEA. As shown in Figure 7b, the flowability and adhesion of sample “f” are increased by 34.1% and 292%, respectively, compared to sample “a”, which means modified UDPEA can interact with flames for a longer period. During this process, active substances such as sodium oxide ions (NaO^−^) and sodium ions (Na^+^) can effectively react with free radicals (H• and •OH) in the flames, thereby terminating the chain reaction of combustion. Modified UDPEA can serve as a cooling medium due to its undecomposed state and its oleophobic properties, which enable it to float on the oil surface and form a thick, dense isolation layer, effectively sealing off flames. Modified UDPEA extinguishes fires through a combination of chemical inhibition, which includes the termination of chain reactions, and physical heat absorption. This results in an extinguishing concentration of 14.183 g/m^3^, which is only 52.7% of the concentration required for UDPEA (26.927 g/m^3^).

By blending FOTS and PFPE in a 1:2 ratio as precursors and uniformly mixing them with TEOS in a liquid phase, hydrolysis and condensation reactions can form a stable transparent sol system in solution. Colloidal particles react with sodium bicarbonate to slowly polymerize into a three-dimensional network structure. The ethyl silane hydroxyl groups on the fluorosiloxane molecules undergo condensation reactions with the hydroxyl groups on the substrate surface and between adjacent ethyl silane hydroxyl groups, forming stable Si-O-Si chemical bonds [29]. One end of the fluorosiloxane molecules anchors to the substrate surface in a cross-linked network structure, while the perfluoroalkyl or fluoroether groups at the other end reduce the surface energy of the substrate. This endows MUDPEA with excellent hydrophobic and oleophobic properties, with hydrophobic and oleophobic angles of 145.169° and 143.542°, respectively. Therefore, when it lands on the oil surface, it does not sink quickly like UDPEA but floats, forming a thermal barrier that isolates oxygen and fuel vapors while cooling the fuel. As a result, it takes 68.5 s of continuous ignition for the aviation kerosene covered with sample “f” to reignite, whereas the kerosene covered with UDPEA reignites instantly upon ignition. Moreover, the flame temperature of the aviation kerosene covered with MUDPEA after re-ignition is significantly lower.

This demonstrates that sample “f” (MUDPEA) outperforms sample “a” (UDPEA) in multiple aspects and has significant fire-extinguishing and anti-reignition effects in practical applications.

## 4. Conclusions

This study investigates the effects of three modification methods (single FOTS, single PFPE, and a combination of FOTS and PFPE at different mass ratios (5:1, 2:1, 1:1, 1:2, 1:5)) on the performance (microstructure, chemical composition, flowability, hydrophobicity and oleophobicity, extinguishing concentration, extinguishing time, and anti-reignition time) of UDPEA based on sodium bicarbonate. The results are as follows:

(1) Modifying UDPEA with either FOTS or PFPE alone improves its water and oil contact angles, but the hydrophobicity and oleophobicity still do not meet the required standards. Additionally, the flowability and fire-extinguishing capability decrease.
(2) The combination of FOTS and PFPE at a 1:2 ratio yields the best results, with the highest water and oil contact angles of 145.169° and 143.542°, respectively. This ratio also achieves the lowest flowability index (*l_p_* = 0.224), minimum extinguishing concentration and time (14.183 g/m^3^ and 1.976 s, respectively), and the longest anti-reignition time of 68.5 s.
(3) When the ratio (FOTS to PFPE) is 1:2, modified UDPEA not only benefits from the endothermic decomposition and asphyxiation of sodium bicarbonate but also has an increased specific surface area and improved flowability. During extinguishing, it effectively absorbs radiative heat and blocks heat transfer, releasing more active substances and accelerating reactions with flame radicals, thereby terminating the combustion chain reaction. The modified UDPEA has an extinguishing concentration of 14.183 g/m^3^, which is only 52.7% of the unmodified UDPEA (26.927 g/m^3^).
(4) The modified UDPEA exhibits excellent hydrophobic and oleophobic properties, floating on the oil surface to form an insulating barrier that isolates oxygen and fuel vapor while cooling the fuel. Its anti-reignition time is 68.5 s, significantly reducing the flame temperature during secondary ignition of aviation kerosene compared to using unmodified UDPEA under the same conditions.

In summary, the modified UDPEA (with a FOTS to PFPE ratio of 1:2) extinguishes fires through a combination of chemical inhibition and physical heat absorption, demonstrating superior environmental friendliness and high performance.

## Data Availability

Dataset available on request from the authors.

## Supplementary Materials

The following supporting information can be downloaded at: https://www.mdpi.com/article/10.3390/molecules29163830/s1, Table S1: Liquidity data for samples a–h.

## Appendix A

**Table A1:** Function of auxiliary ingredient of SHOU DPEA.

Auxiliary Ingredient	Function
mica	It acts as a functional filler, providing reinforcement and enhancing the mechanical properties of the powder.
talc	It functions as a lubricant, improving flowability and preventing caking or agglomeration of the powder particles.
activated clay	It acts as a rheology modifier, controlling the viscosity and thixotropic properties of the powder suspension.
zeolite	It serves as an adsorbent, removing impurities or unwanted gases from the system and improving powder stability.
nano calcium carbonate	It functions as a reinforcing agent, enhancing the strength and stiffness of the powder material.
Silicone Oil	It acts as a surface modifier, reducing friction between powder particles and improving flowability.
C6-fluorinated acrylate copolymer	(1) C6-fluorinated acrylate copolymer can form a thin and uniform wetting layer on the surface of ultrafine dry powder, improving compatibility and adhesion with other materials such as coatings or adhesives. (2) Due to its hydrophobic properties, C6-fluorinated acrylate copolymer can create a protective layer on the surface of UDPEA, enhancing the longevity of the powder. (3) The low surface energy of C6-fluorinated acrylate copolymer reduces the adhesive forces between particles of UDPEA, preventing agglomeration and caking, and improving flowability and dispersibility.
