# Supplementary material for: Modification and Application Performance Study of Ultra-Fine Dry Powder Extinguishing Agent
Source: Molecules. 2024 Aug 12;29(16):3830. doi: 10.3390/molecules29163830 (PMC11357566; doi:10.3390/molecules29163830)
Supplement: Supplementary file 1 [file molecules-29-03830-s001.zip › molecules-3109859-supplementary.pdf]

Table S1. Liquidity data for samples a-h

N	a	b	c	d	e	f	g	h
50	191.67	375.00	452.78	542.86	604.17	483.33	257.14	591.67
100	302.63	500.00	525.81	584.62	690.48	580.00	439.02	525.93
150	453.95	562.50	660.81	712.50	776.79	750.00	627.91	591.67
200	605.26	666.67	795.12	894.12	935.48	966.67	800.00	747.37
250	756.58	833.33	993.90	1027.03	1098.48	1208.33	1000.00	887.50
300	896.10	900.00	1164.29	1232.43	1318.18	1450.00	1200.00	1065.00
350	1045.45	1050.00	1358.33	1437.84	1537.88	1691.67	1400.00	1242.50
400	1194.81	1200.00	1552.38	1643.24	1757.58	1933.33	1600.00	1420.00
450	1344.16	1350.00	1746.43	1848.65	1977.27	2175.00	1800.00	1597.50
500	1493.51	1500.00	1940.48	2054.05	2196.97	2416.67	2000.00	1775.00
slope	2.93	2.48	3.42	3.51	3.67	4.47	3.89	2.90
Intercept	22.69	211.11	177.32	233.09	280.21	135.56	43.31	245.89
l_p	0.34	0.40	0.29	0.29	0.27	0.22	0.26	0.34
f_a	7.74	85.05	51.78	66.45	76.36	30.31	11.14	84.68
R^2	1.000	0.995	0.995	0.993	0.990	0.997	1.000	0.980
